# Increased prevalence but decreased survival of nonviral hepatocellular carcinoma compared to viral hepatocellular carcinoma in recent ten years

**DOI:** 10.1038/s41598-024-59668-2

**Published:** 2024-04-20

**Authors:** Ting-Chun Chen, Shun-Wen Hsiao, Yang-Yuan Chen, Hsu-Heng Yen, Wei-Wen Su, Yu-Chun Hsu, Siou-Ping Huang, Pei-Yuan Su

**Affiliations:** 1 Division of Endocrinology and Metabolism, Cheng Ching Hospital, Taichung, Taiwan; 2 Division of Gastroenterology, Changhua Christian Hospital, Changhua, Taiwan; 3 Department of Post-Baccalaureate Medicine, College of Medicine, National Chung Hsing University, Taichung, Taiwan; 4 Division of Gastroenterology, Yuanlin Christian Hospital, Changhua, Taiwan; 5 Department of Hospitality Management, MingDao University, Changhua, Taiwan

**Keywords:** Viral hepatocellular carcinoma, Non-viral hepatocellular carcinoma, Type 2 diabetes mellitus, Non alcoholic fatty liver disease, Alcoholic liver disease, Cancer, Gastroenterology

## Abstract

Due to the comprehensive hepatitis B virus vaccination program in Taiwan since 1986, the development of antiviral therapy for chronic hepatitis B and chronic hepatitis C infection and covered by National health insurance. Besides, the increased prevalence of nonalcoholic fatty liver disease (NAFLD) and currently, approved therapy for NAFLD remain developing. The etiology of liver-related diseases such as cirrhosis and hepatocellular carcinoma required reinterpretation. This study aimed to analyze the incidence and outcome of hepatocellular carcinoma (HCC) due to viral (hepatitis B and hepatitis C) infection compared to that of nonviral etiology. We retrospectively analyzed patients with HCC from January 2011 to December 2020 from the cancer registry at our institution. Viral-related hepatitis was defined as hepatitis B surface antigen positivity or anti-hepatitis C virus (HCV) antibody positivity. A total of 2748 patients with HCC were enrolled, of which 2188 had viral-related HCC and 560 had nonviral-related HCC. In viral HCC group, the median age at diagnosis was significantly lower (65 years versus 71 years, *p* < 0.001), and the prevalence of early-stage HCC, including stage 0 and stage A Barcelona Clinic Liver Cancer, was significantly higher (52.9% versus 33.6%, *p* < 0.001). In nonviral HCC group, alcohol use was more common (39.9% versus 30.1%, *p* < 0.001), the prevalence of type 2 diabetes mellitus (T2DM) was higher (54.5% versus 35.1%, *p* < 0.001), and obesity was common (25.0% versus 20.5%, *p* = 0.026). The prevalence of nonviral HCC increased significantly from 19.2 to 19.3% and 23.0% in the last 10 years (*p* = 0.046). Overall survival was better in the viral HCC group (5.95 years versus 4.00 years, *p* < 0.001). In the early stage of HCC, overall survival was still better in the viral HCC group (*p* < 0.001). The prevalence of nonviral HCC has significantly increased in the last ten years. The overall survival was significantly lower in the nonviral HCC, perhaps because the rate of early HCC detection is lower in nonviral HCC and anti-viral therapy. To detect nonviral HCC early, we should evaluate liver fibrosis in high-risk groups (including people with obesity or T2DM with NAFLD/NASH and alcoholic liver disease) and regular follow-up for those with liver fibrosis, regardless of cirrhosis.

## Introduction

Hepatocellular carcinoma (HCC) was the sixth most commonly diagnosed malignancy and the third primary cause of cancer-related mortality worldwide in 2020^1^. The major risk factors for HCC include chronic viral hepatitis, including infection with the hepatitis B virus (HBV) and the hepatitis C virus (HCV), heavy alcohol use, metabolic liver disease especially nonalcoholic fatty liver disease (NAFLD), type 2 diabetes mellitus (T2DM), and exposure to toxins such as aflatoxins^2^.

In Taiwan, chronic HBV and HCV are high-prevalence diseases, with reported rates of 20% and 1.8–5.5% of the general population, respectively, and are major causes of HCC^3–5^. We implemented the first comprehensive neonatal HBV vaccination program to prevent mother-to-child transmission in 1986^6^. Diagnostic abdominal ultrasonography was initiated and covered by National Health Insurance (NHI) for high-risk groups, namely people with HBV infection, HCV infection, and cirrhosis^7^. Therapy for chronic HBV has been covered by NHI since 2003. Direct-acting antivirals (DAAs) have also been proven to reduce the rate of HCV related HCC development and recurrence after achieving sustained virological response^8^. The Taiwan National Hepatitis C Program Office was established in 2016, and NHI enrolled DAAs therapy for HCV since 2017. Thus, the burden of viral related end-stage liver disease, such as HCC, and cirrhosis, was decreased^9^ Besides, with the report of the concomitantly increased prevalence of MAFLD-related HCC and the growing obesity epidemic, it is crucial to address the challenges posed by nonviral HCC^10^ In this study, we analyze the changes in the etiology of patients with HCC throughout the last 10 years (2011–2020) and compare them with metabolic factors such as body mass index (BMI), obesity, and T2DM.

## Methods

### Inclusion criteria

We retrospectively analyzed the records of patients diagnosed with novel HCC from January 2011 to December 2020 obtained from the cancer registry center at our institution. All methods were performed in accordance with relevant guidelines and regulations. The informed consent was waived due to minimal risk and approved by the Ethics Committee of Changhua Christian Hospital. Patients who did not meet the diagnostic imaging criteria required biopsy with histopathology diagnosis. A staging system was applied on the update of the Barcelona Clinic Liver Cancer (BCLC) stage into very early stage (0): single tumor ≤ 2 cm, early stage (A): single tumor or multifocal HCC up to three nodules each measuring ≤ 3 cm, intermediate stage (B): multiple nodules, and advanced stage (C): tumors with portal invasion with or without extrahepatic spread, or performance status (PS) 1 or 2, terminal stage (D): any tumor burden with end-stage liver function, Child–Pugh C, or PS 3, or 4^11^. PS was classified by the Eastern Cooperative Oncology Group (ECOG) into 0, 1, 2, 3, and 4. Viral hepatitis (including the HBV infection and the HCV infection) was defined as hepatitis B surface antigen positivity or anti-HCV antibody positivity. The variables extracted from medical records included age, sex, HCC staging, BMI, laboratory measurements, and comorbidities such as T2DM.

### Statistical analysis

The one-sample Kolmogorov–Smirnov test was used to verify the normality of data distribution. Continuous data are presented as mean values and standard deviations (for normally distributed data) or median values with interquartile ranges (for skewed data). Categorical data are presented as frequencies and percentages. Comparisons between the viral HCC and nonviral HCC groups were performed using the chi-square test for categorical variables and the Mann–Whitney U-test for continuous variables. The time at risk was measured from the date of HCC diagnosis until the last follow-up date or death, whichever came first. Survival curves were plotted via the Kaplan–Meier method, and the differences in survival status between groups were compared using the log-rank test. The association between clinical factors and survival was evaluated using Cox proportional hazards models in univariate and multivariate analyses. The factors that were significantly associated in univariate analyses were entered into the multivariate adjustment model via backward elimination. The risk was expressed as hazard ratios (HRs) and 95% confidence intervals (CIs). All tests were two-sided, and *p*-values < 0.05 were considered statistically significant. Statistical analyses were performed using IBM SPSS version 22.0 (IBM Corp., Armonk, NY).

### Ethical approval and consent to participate

The Ethics Committee of Changhua Christian Hospital approved the study protocol used in this study (CCH IRB No: 220327).

## Results

### Baseline characteristics

A total of 2748 patients with HCC were enrolled as shown in Table 1. Among these, there were 2188 with viral-related HCC and 560 with nonviral-related HCC. The median diagnosed age was significantly lower in the viral HCC group (65.2 years vs. 69.6 years, *P* < 0.001). Early-stage HCC, including BCLC stage 0, and stage A, was significantly higher (in the viral HCC group (52.9% vs. 33.6%, *P* < 0.001)). Next, 55.0% of people in the viral HCC group and 46.5% of those in the nonviral HCC group had cirrhosis. The proportion of people with preserved liver function, Child–Pugh A, was significantly higher in the viral HCC group (73.1% vs. 57.2%, *P* < 0.001). T2DM was significantly higher in the nonviral (HCC group (54.5% vs. 35.1%, *P* < 0.001)). Obesity, BMI ≥ 27.5 kg/m^2^, was significantly higher in the nonviral HCC group (25.0% vs. 20.5%, *p* = 0.026). Alcohol use was significantly higher in the nonviral (HCC group (39.9% vs. 30.1%, *P* < 0.001)). There were no statistically significant differences in sex and BMI between the two groups.

**Table 1:** Characteristics of the patients with viral HCC and nonviral HCC.

Variables	ALL Patients	Viral HCC	Nonviral HCC	*p*-value
	n = 2748	n = 2188	n = 560	
Age, years, Median (IQR)	66 (59–75)	65 (58–73)	71 (62–78)	< 0.001
Sex (M), n (%)	1996 (72.6)	1583 (72.3)	413 (73.8)	0.507
BMI, kg/m^2^, Median (IQR)	24.24 (21.79–26.93)	24.22 (21.77–26.81)	24.38 (21.97–27.49)	0.359
Obesity (BMI ≧27.5), n (%)	580 (21.4%)	442 (20.5%)	138 (25.0%)	0.026
Cirrhosis	1441 (53.3)	1185 (55.0)	256 (46.5)	< 0.001
BCLC, n (%)				< 0.001
0 + A	1345 (48.9)	1157 (52.9)	188 (33.6)	< 0.001
B	610 (22.2)	453 (20.7)	157 (28.0)	< 0.001
C	560 (20.4)	413 (18.9)	147 (26.3)	< 0.001
D	233 (8.5)	165 (7.5)	68 (12.1)	< 0.001
α-FP > 400, n (%)	653 (24.8)	504 (24.0)	149 (28.3)	0.041
Child–Pugh score, n (%)				< 0.001
A	1876 (69.9)	1567 (73.1)	309 (57.2)	< 0.001
B	624 (23.2)	441 (20.6)	183 (33.9)	< 0.001
C	184 (6.9)	136 (6.3)	48 (8.9)	0.046
ECOG ≥ 3, n (%)	376 (13.8)	271 (12.4)	105 (18.9)	< 0.001
Smoking, n (%)	1119 (40.8)	868 (39.7)	251 (44.9)	0.026
Alcohol, n (%)	880 (32.1)	657 (30.1)	223 (39.9)	< 0.001
T2DM, n (%)	1073 (39.0)	768 (35.1)	305 (54.5)	< 0.001

*BMI* body mass index, *BCLC* Barcelona Clinic Liver Cancer classification, *α-FP* α-fetoprotein, *ECOG* Eastern Cooperative Oncology Group performance status, *T2DM* type 2 diabetes mellitus.

### Baseline characteristics between different periods of time

We analyzed the characteristics between different periods of time as shown in Table 2. In the period from 2018 to 2020, we found older age (65 years vs. 66 years vs. 67 years, *p* = 0.049), higher BMI (23.88 kg/m^2^ vs. 24.11 kg/m^2^ vs. 24.59 kg/m^2^, *p* = 0.009), more preserved liver function (Child–Pugh A, 66.3% vs. 68.7% vs. 74.9%, *p* < 0.001), better performance status (ECOG < 2, 67.9% vs. 91.8% vs. 96.4%, *p* < 0.001), and a higher percentage of anti-viral therapy for HBV (43.8% vs. 57.8% vs. 62.2%, *p* < 0.001).

**Table 2:** Characteristics of the patients in different periods of time.

Variables	ALL Patients	The different periods of HCC diagnosis			
		2011–2013	2014–2017	2018–2020	*p*-value
	n = 2748	n = 797	n = 1111	n = 840	
Age, years, Median (IQR)	66 (59–75)	65 (58–74)	66 (58–75)	67 (60–76)	0.049
Sex (M), n (%)	1996 (72.6)	561 (70.4)	825 (74.3)	610 (72.6)	0.174
BMI, kg/m^2^, Median (IQR)	24.24 (21.79–26.93)	23.88 (21.61–26.58)	24.11 (21.78–27.08)	24.59 (22.07–27.13)	0.009
Obesity (BMI ≧27.5), n (%)	580 (21.4)	153 (19.5)	239 (21.9)	188 (22.6)	0.273
Cirrhosis	1441 (53.3)	434 (55.2)	585 (54.1)	422 (50.4)	0.122
BCLC, n (%)					0.333
0 + A	1345 (48.9)	386 (48.4)	554 (49.9)	405 (48.2)	
B	610 (22.2)	170 (21.3)	240 (21.6)	200 (23.8)	
C	560 (20.4)	163 (20.5)	219 (19.7)	178 (21.2)	
D	233 (8.5)	78 (9.8)	98 (8.8)	57 (6.8)	
α-FP > 400, n (%)	653 (24.8)	199 (26.4)	254 (23.8)	200 (24.8)	0.452
Child–Pugh score, n (%)					0.002
A	1876 (69.9)	507 (66.3)	749 (68.7)	620 (74.9)	< 0.001
B	624 (23.2)	199 (26.0)	257 (23.6)	168 (20.3)	0.025
C	184 (6.9)	59 (7.7)	85 (7.8)	40 (4.8)	0.021
ECOG ≥ 3, n (%)	376 (13.8)	256 (32.1)	90 (8.2)	30 (3.6)	< 0.001
Smoking, n (%)	1119 (40.8)	221 (27.9)	487 (43.8)	411 (48.9)	< 0.001
Alcohol, n (%)	880 (32.1)	186 (23.5)	380 (34.2)	314 (37.4)	< 0.001
T2DM, n (%)	1073 (39.0)	294 (36.9)	441 (39.7)	338 (40.2)	0.324
HBV with antiviral therapy, n (%)	625 (55.2)	141 (43.8)	268 (57.8)	216 (62.2)	< 0.001

*BMI* body mass index, *BCLC* Barcelona Clinic Liver Cancer classification, *α-FP* α-fetoprotein, *ECOG* Eastern Cooperative Oncology Group performance status, *T2DM* type 2 diabetes mellitus.

### Factors associated with mortality

The determinants of survival are shown in Table 3. They include age, BMI, the viral cause of HCC, early stage of BCLC, a high alpha-fetoprotein level (> 400), preserved liver function (Child–Pugh A), and good performance (ECOG < 2). Those factors were also statistically significant in the multivariate analysis. We also evaluated their influence on overall survival in T2DM; however, the factors were not statistically significant in the multivariate analysis.

**Table 3:** Univariate and multivariate analyses for factors affecting survival in HCC.

Variables	Univariate analysis		Multivariate analysis	
	HR (95% CI)	*p-*value	HR (95% CI)	*p-*value
Age, years	1.02 (1.01–1.02)	< 0.001	1.02 (1.02–1.03)	< 0.001
Sex (Male)	1.15 (1.02–1.3)	0.022	1.24 (1.08–1.41)	0.002
BMI, kg/m^2^	0.94 (0.93–0.96)	< 0.001	0.98 (0.96–0.99)	0.002
Etiology				
Nonviral HCC	1 (Reference)	–	1 (Reference)	–
Viral HCC	0.61 (0.54–0.68)	< 0.001	0.74 (0.64–0.84)	< 0.001
BCLC				
0 + A	1 (Reference)	–	1 (Reference)	–
B	1.93 (1.67–2.25)	< 0.001	1.67 (1.42–1.96)	< 0.001
C	6.65 (5.8–7.63)	< 0.001	4.05 (3.44–4.77)	< 0.001
D	11.55 (9.75–13.68)	< 0.001	4.69 (3.35–6.58)	< 0.001
AFP > 400	3.15 (2.81–3.53)	< 0.001	1.79 (1.57–2.03)	< 0.001
Child				
A	1 (Reference)	–	1 (Reference)	–
B	3.27 (2.91–3.68)	< 0.001	1.8 (1.57–2.06)	< 0.001
C	7.32 (6.15–8.71)	< 0.001	1.78 (1.25–2.53)	0.001
ECOG ≥ 3	12.12 (10.49–14)	< 0.001	5.07 (4.3–5.98)	< 0.001
Smoking	1.07 (0.96–1.19)	0.22	–	–
Alcohol	1.02 (0.91–1.14)	0.795	–	–
T2DM	0.84 (0.75–0.93)	0.002	–	–

*BMI* body mass index, *BCLC* Barcelona Clinic Liver Cancer classification, *α-FP* α-fetoprotein, *ECOG* Eastern Cooperative Oncology Group performance status, *T2DM* type 2 diabetes mellitus.

### Prevalence of viral and nonviral HCC in different time periods

**Figure 1:**
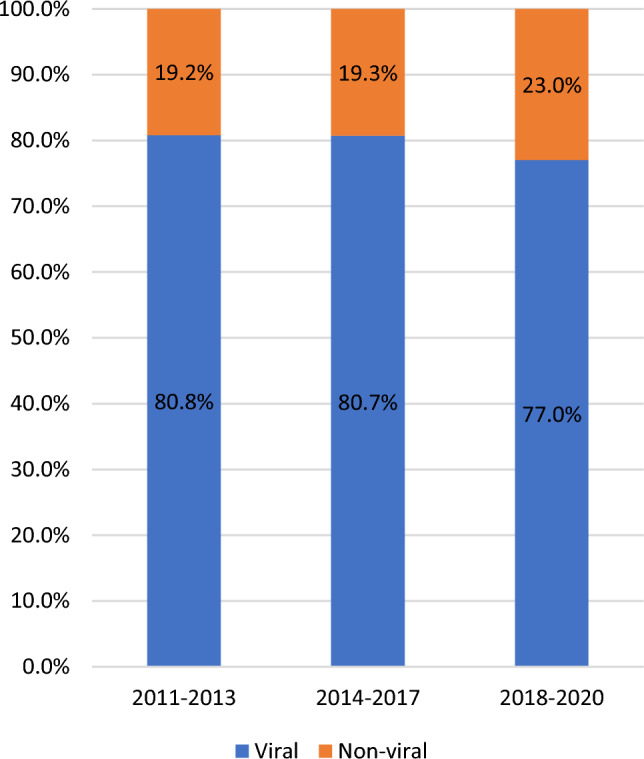
Comparison between viral HCC and nonviral HCC in recent years. The prevalence of nonviral HCC increased significantly from 19.2% in 2011–2013 and 19.3% in 2014–2017 to 23.0% in 2018–2020 (*P* = 0.046).

During the last 10 years (Fig. 1), compared with viral HCC, the prevalence of nonviral HCC increased gradually from 19.2% in 2011–2013 to 19.3% in 2014–2017 and significantly increased to 23.0% in 2018–2020 (*P* = 0.046).

### Overall survival in different time periods

**Figure 2:**
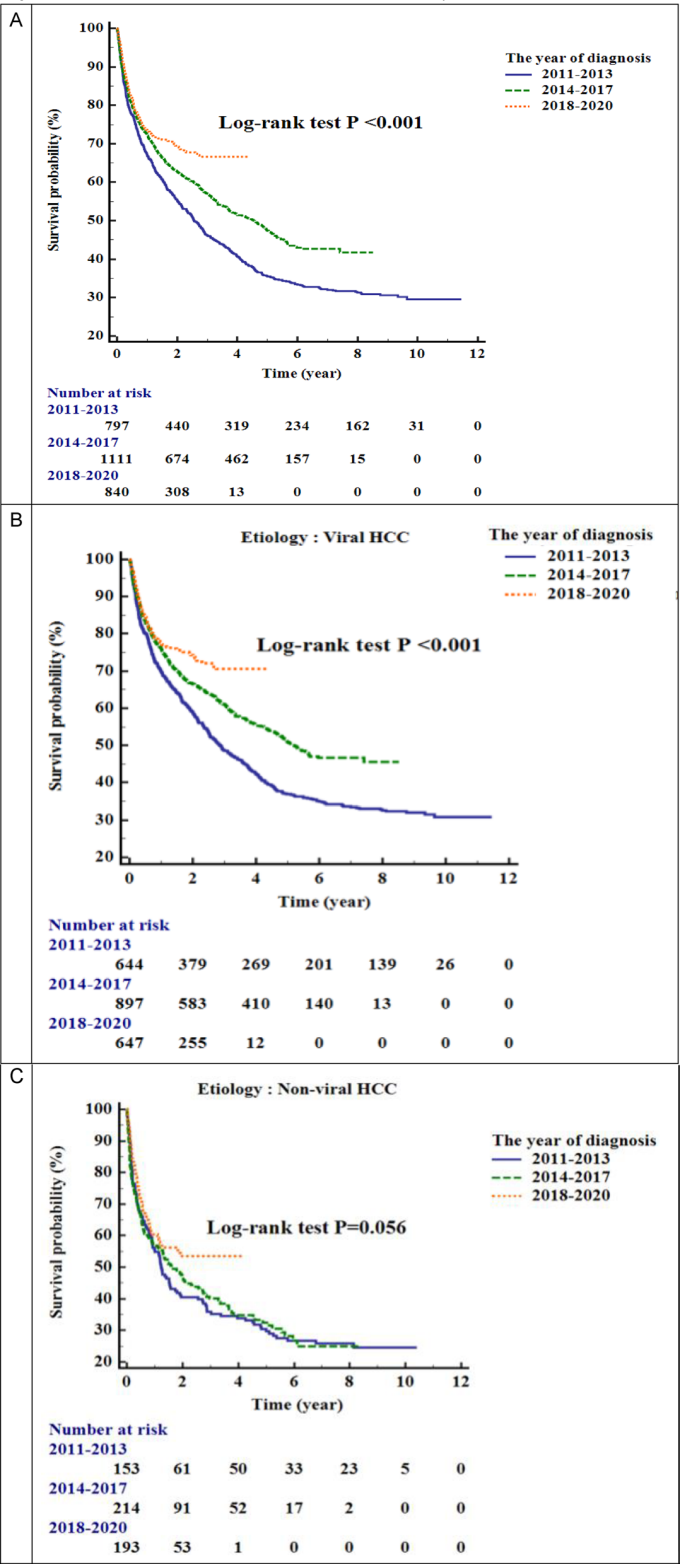
Comparisons of overall survival in recent years. (**A**) The median survival was not attained in 2018–2020, which was better than those in 2014–2017 (median survival: 4.54 years) and 2011–2013 (median survival: 2.56 years) (*P* < 0.001). (**B**) For viral HCC, the median survival was not attained in 2018–2020, which was better than those in 2014–2017 (median survival: 5.21 years) and 2011–2013 (overall survival: 2.86 years) (*P* < 0.001). (**C**) For nonviral HCC, the median overall survival did not differ significantly between the different periods (*P* = 0.056).

We analyzed the overall survival for different periods and presented the results in Fig. 2. Figure 2A compared with different periods, the median survival was not attained in 2018–2020, which was better than that in 2014–2017 (median survival of 4.54 years) and in 2011–2013 (median survival of 2.56 years) (*P* < 0.001). We analyzed viral HCC in different periods, showed in Fig. 2B, and found that the overall survival was not attained in 2018–2020, which was better than that in 2014–2017 (overall survival of 5.21 years) and in 2011–2013 (overall survival of 2.86 years) (*P* < 0.001). However, in nonviral HCC, showed in Fig. 2C, the overall survival did not differ significantly in different periods (*P* = 0.056).

### Overall survival in viral and nonviral HCC

**Figure 3:**
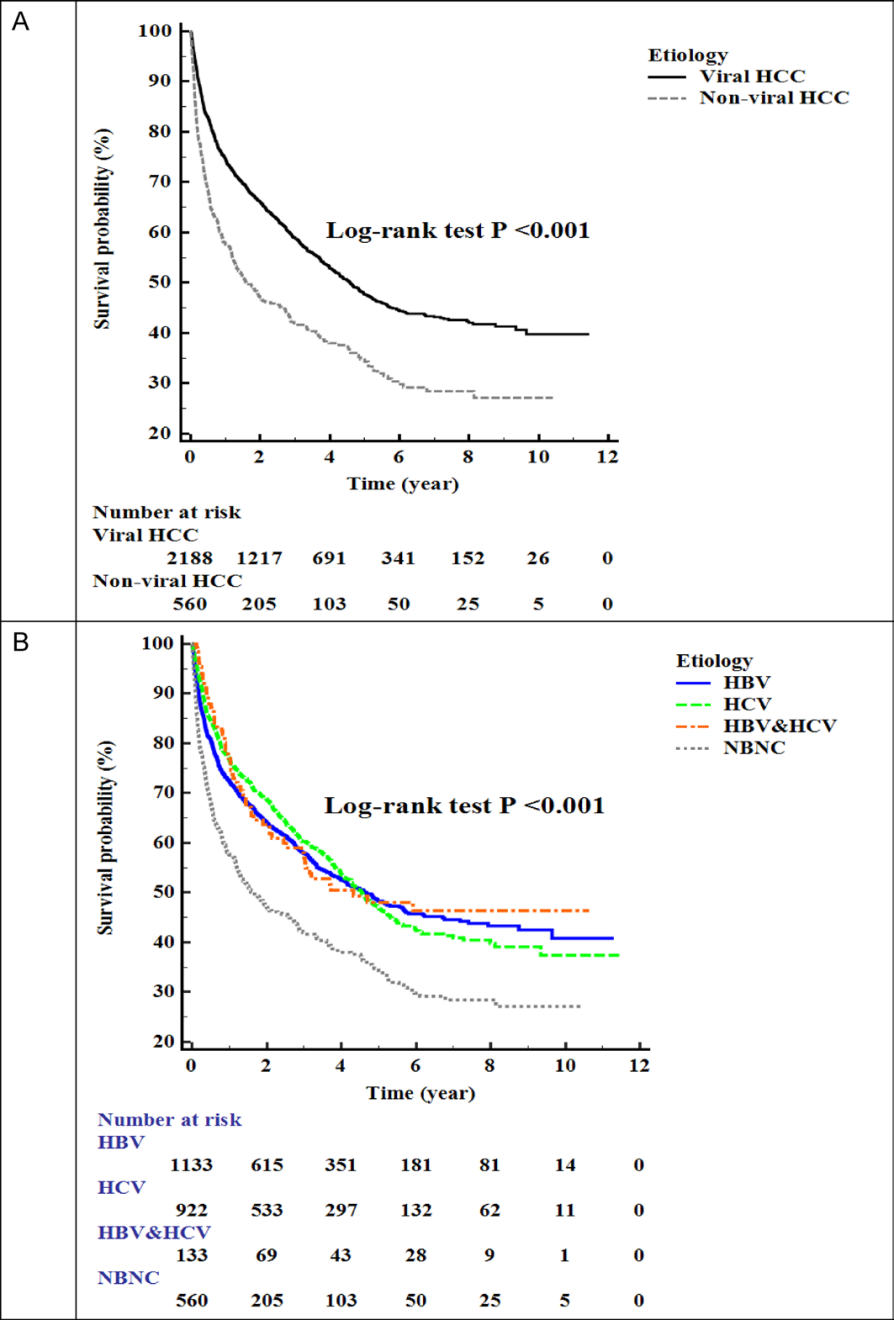
Comparison of the overall survival between viral and nonviral HCC. (**A**) Viral HCC vs. nonviral HCC. The median survival durations in viral HCC and nonviral HCC were 4.58 years and 1.60 years, respectively, and there was no statistically significant difference between them (*p* < 0.001). (**B**) HBV, HCV, both HBV and HCV, nonviral HCC. We divided viral causes of HCC into HBV, HCV, and both HBV, and HCV. The median survival was better in HBV, HCV, and both HBV, and HCV (4.67 years, 4.55 years, 4.33 years, respectively) than in nonviral HCC (1.60 years) with each of the differences being statistically significant (all *P* < 0.001).

We compared overall survival in the viral and nonviral HCC, Fig. 3A, and found that the median overall survival values in these groups were 4.58 years and 1.60 years, respectively, and the difference between the two values was statistically significant (*P* < 0.001). In Fig. 3B, we divided viral etiologies of HCC into HBV, HCV, and both HBV, and HCV. The median survival was better with HBV, HCV, and both HBV, and HCV (4.67 years, 4.55 years,4.33 years, respectively) etiologies than with nonviral HCC (1.60 years), with all differences being statistically significant (*P* < 0.001).

### Overall survival according to BCLC stage

**Figure 4:**
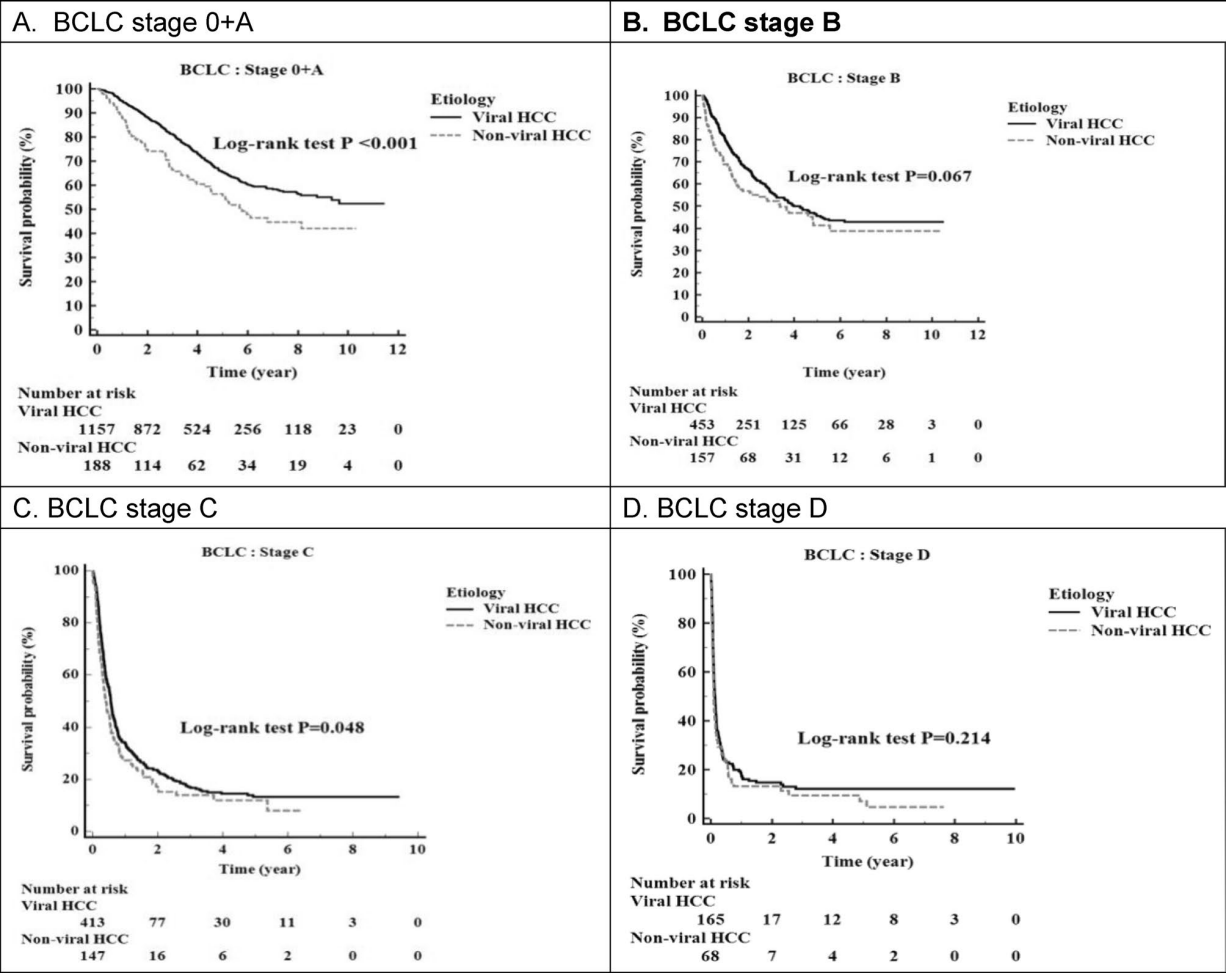
Comparison of early-stage survival between viral and nonviral HCC. (**A**) In early-stage HCC, namely BCLC stage 0, and stage A, the median survival was not attained in viral HCC and was 5.77 years in nonviral HCC. This parameter was significantly better in viral HCC (*P* < 0.001). (**B**) In BCLC stage B, the median survival was 4.25 years in viral HCC and 3.73 years in nonviral HCC (*P* = 0.067). (**C**) In BCLC stage C, the median survival was 0.56 years in viral HCC and 0.41 years in nonviral HCC (*P* = 0.048). (**D**) In BCLC stage D, the median survival was 0.14 years in viral HCC and 0.10 years in nonviral HCC (*P* = 0.214).

We also evaluated the overall survival according to the BCLC stage. Figure 4A showed early-stage HCC, namely BCLC stage 0, and stage A, the median survival was not attained in viral HCC and was 5.77 years in nonviral HCC, which was significantly better in viral HCC (*P* < 0.001). In BCLC stage B, Fig. 4B, the median survival values in viral HCC and nonviral HCC were 4.25 years 3.73 years, respectively (*p* = 0.067). In BCLC stage C, Fig. 4C, the median survival was 0.56 years in viral HCC and 0.41 years in nonviral HCC (*p* = 0.048). In BCLC stage D, Fig. 4D, the median survival was 0.14 years in viral HCC and 0.10 years in nonviral HCC (*p* = 0.214).The overall survival in viral HCC and nonviral HCC did not differ significantly in BCLC stage B, C, or D.

## Discussion

In our cohort, the prevalence of nonviral HCC increased significantly in recent years. More preserved liver function, Child–Pugh A, and an early stage of HCC at diagnosis were found in viral HCC. These may reflect in the overall survival, which was also better in the viral HCC group. Determinants of overall survival included age, BMI, the viral cause of HCC, early stage of BCLC, high alpha-fetoprotein level (> 400), preserved liver function (Child–Pugh A), and good performance (ECOG < 2).

Apart from comprehensive HBV vaccinations, we provided antiviral therapy since 2003 for specific condition, and for cirrhosis since 2010. We expand the payment condition with time. With the development of direct-acting antivirals, the foundation of the National Hepatitis C Program Office, we provided antiviral therapy for chronic hepatitis C with viremia since 2017. The burden of viral related end-stage liver disease, together with its complications, and related mortality, has been reported to decrease previously^9,12^. Thus, in our study, more percentage of HBV related HCC received antiviral therapy in recent years, and viral HCC was found to have a more preserved liver function and was diagnosed at an earlier stage.

T2DM influences HCC mortality. A recent meta-analysis including 21 studies and 9767 patients with was conducted to evaluate DM and the prognosis of HCC^13^. The adjusted HRs were 1.55 (95% CI 1.27–1.91; *P* < 0.001) for overall survival and 2.15 (95% CI 1.75–2.63; *P* < 0.001) for disease-free survival, indicating DM was an independent determinant of a poor prognosis in HCC for both overall survival and disease-free survival. However, some studies revealed the significance of the impact of diabetes on the prognosis of HCC only in the subgroup setting. In a meta-analysis involving 10 studies, an analysis of the prognosis of HCC in patients with DM after curative treatments revealed a poor prognosis in patients with DM and HCC only for tumors measuring ≤ 5 cm but no influence for tumors measuring > 5 cm with HRs of 1.63 (95% CI 1.25–2.12) and 0.67(95% CI 0.39–1.15), respectively^14^. In a retrospective study conducted in Taiwan, metformin use was shown to decrease the five-year cumulative rate of HCC development from 10.9 to 2.6% in T2DM after successful antiviral therapy for HCV infection (aHR: 2.83; 95% CI 1.57–5.08) In our study, we demonstrated a significantly increased incidence of nonviral HCC in recent years. Up to 54.5% of patients with nonviral HCC had T2DM. However, the impact of HCC on the prognosis of T2DM was not statistically significant in the multivariate analysis.

It is difficult to identify the correlation between nonviral HCC and NAFLD. NAFLD-related HCC still needs to be thoroughly investigated. The surveillance strategy was uncertain and difficult in NAFLD-related HCC^15^. In our cohort, 53.5% of the patients in the nonviral HCC group did not have liver cirrhosis. Despite the lower annual incidence in the NAFLD-related (HCC group (0.44 per 1000 person-years)), several countries, including nations in Europe and Asia, have shown increased prevalence rates of nonviral HCC and its association with NAFLD^10,16,17^. There was strong evidence supporting abdominal ultrasound screening to reduce HCC mortality^12,13^. Meanwhile, owing to the comprehensive surveillance program, we can detect very early or early stages during which it is preferable for the patient to receive curative therapies, which would also reflect on overall survival^2^.However, NAFLD without cirrhosis was estimated to be less than 1.5% per year and inefficient for surveillance^15^.

In patients with NAFLD or NASH, weight reduction may improve liver steatosis, inflammation, and fibrosis; however, there is insufficient data on the reduction of the incidence of HCC^18^. In a European cohort study involving 467,336 cases, reporting more than two hours of vigorous physical activity per week was associated with a reduced risk of liver cancer development compared with reporting no vigorous physical activity (HR: 0.50; 95% CI 0.33–0.76), and adhering to a Mediterranean diet also seemed to decrease HCC risk^19^^20^. In our study, we noticed that even in early-stage HCC, nonviral HCC was associated with a poor prognosis. One plausible explanation is the lack of reversal of hepatic impairment associated with nonviral HCC.

Another eminent cause of nonviral HCC was ALD. In a meta-analysis on alcoholic cirrhosis, the cumulative incidence rates of HCC development in five years and ten years of follow-up were 3% and 9%, respectively^21^. Among patients with alcohol-related cirrhosis, alcohol abstinence reduced the risk of HCC but without a history of a decompensated event^22^. Thus, not only surveillance in ALD-related cirrhosis but active engagement in abstinence programs for patients with alcohol use disorders is also crucial.

Nevertheless, our study had several limitations. First, it was a single-center study with a limited sample size. Second, being a retrospective study, there were some missing data on comorbidities, metabolic factors, and the proportion of NAFLD/NASH, and ALD. Third, we did not take the treatment strategy for HCC into consideration, and this affected survival data. Although the prevalence of nonviral HCC is increasing, further research is needed.

## Conclusions

The prevalence of nonviral HCC has increased significantly in recent years; however, it shows a poorer prognosis than viral HCC. Our results highlight the need to investigate nonviral-related liver diseases, especially NAFLD and ALD. High-risk groups such as patients with T2DM or metabolic syndrome should be included in surveillance programs. Although no solid conclusions regarding surveillance strategies were drawn, patients with significant liver fibrosis, regardless of cirrhosis, should be referred to hepatologists for rigorous and regular surveillance.

## Data Availability

The datasets are available and uploaded as related files.
